# CHILDHOOD SPEECH IMPAIRMENT AND DEMENTIA RISKS AMONG US OLDER ADULTS

**DOI:** 10.1093/geroni/igae098.4140

**Published:** 2024-12-31

**Authors:** Haowei Wang, Shu Xu, Yalian Pei

**Affiliations:** Syracuse University, Syracuse, New York, United States; University of Michigan, Ann Arbor, Michigan, United States; Syracuse University, Syracuse, New York, United States

## Abstract

Speech problems in childhood have profound implications for learning, communication, as well as the development of social and cognitive skills in adulthood. However, research has yet examined how early life speech problems may impact subsequent dementia risks in later life. Using a nationally representative data from the longitudinal Health and Retirement Study, this study investigated how the experience of speech problems before age 16 was associated with the risk of dementia among older adults aged 50 and older. We pooled the full life history information about childhood speech problems for N = 19,082 participants who had 1-11 observations for cognitive and sociodemographic information from 2000 to 2020. We constructed person-interval data and estimated discrete-time event history models to examine risks for developing dementia, which was measured using a validated Langa-Weir classification of cognitive function. We found that about 3.45% of US older adults had speech problems in childhood, with a higher prevalence for younger cohorts, male, and non-Hispanic White, compared to their counterparts. Results showed that older adults with childhood speech problems had higher risks of developing dementia in later life (RRR = 1.49, p < 0.01), compared to those who never had speech problems in childhood. The effects of childhood speech problem on dementia risks were partially explained by BMI, activities of daily living (ADL) limitations, and cardiometabolic and depressive symptoms. Findings highlight the long-arm impact of early life health adversity and suggest the needs for screening and early intervention programs for childhood speech impairment.

